# Exploring the Impact of Protein Supplement Source on Body Composition in Women Practicing Anaerobic Resistance Exercise: A Pilot Study

**DOI:** 10.3390/nu16020321

**Published:** 2024-01-22

**Authors:** Juan Manuel Ballesteros-Torres, Anayansi Escalante-Aburto, María Elena Villarreal-Arce, Cindy Joanna Caballero-Prado

**Affiliations:** 1 Departamento de Microbiología e Inmunología, Facultad de Ciencias Biológicas (FCB), Universidad Autónoma de Nuevo León (UANL), Ave. Universidad s/n, Cd. Universitaria, San Nicolás de los Garza 66450, Mexico; juan.ballesterostr@uanl.edu.mx; 2 Tecnologico de Monterrey, Institute for Obesity Research, Eugenio Garza Sada 2501, Monterrey 64849, Mexico; 3 Departamento de Nutrición, Universidad de Monterrey (UDEM), Escuela de Ciencias Aliadas a la Salud, Ave. Ignacio Morones Prieto, 4500 Pte., San Pedro Garza García 66238, Mexico; maria.villarreala@udem.edu

**Keywords:** protein supplements, amino acids, body composition, body mass index, bioelectrical impedance, resistance exercise, muscle mass, Mexican woman

## Abstract

Supplements based on protein hydrolysates have been used as an effective source to access amino acids with greater bioavailability, promoting absorption to improve body composition. Five groups of young women were randomly selected. They followed a personalized eating plan that included different protein supplements (meat, vegan, branched-chain amino acids [BCAAs], whey, and control group), combined with an exercise plan, for eight weeks, aiming to assess their consumption effects combined with resistance exercise on body composition. Bioelectrical impedance before and after the treatment was conducted. The results showed that the supplementation with BCAAs presented a significant decrease (*p* < 0.05) on the BMI in this group (initial BMI = 19.7 kg/m^2^; final BMI = 19.4 kg/m^2^). When comparing the final measures among the groups, the BCAAs and vegan supplements caused a significant decrease in body weight (50.24 kg and 51.34 kg, respectively). The BMI of the group supplemented with meat proteins was statistically higher (22.06 kg/m^2^) than that the group supplemented with BCAAs (19.4 kg/m^2^) (*p* < 0.05). No significant changes were observed in the type of protein consumed to produce muscle mass in the participants after eight weeks of study under a controlled diet and anaerobic resistance exercise. Participants exhibited energy deficiencies, but their macronutrient distribution appeared normal. Following an 8-week intervention, meat and BCAAs reduced weight and BMI, although no statistical differences were observed. It is recommended to extend the treatment for a more comprehensive understanding.

## 1. Introduction

The confluence of protein-based dietary supplements and resistance training significantly impact muscular physiology, particularly regarding enhancing muscle mass and strength. In alternative scenarios, this combination offers a pivotal strategy to mitigate the potential onset of sarcopenia [1]. Nevertheless, numerous factors, including age, gender, exercise modality, and the quantity and composition of dietary supplements ingested, can exert diverse effects on these physiological processes, yielding a spectrum of outcomes [2]. Resistance exercise influences skeletal muscle, focusing on two fundamental processes: (1) the synthesis of muscle proteins and (2) the degradation of muscle proteins. It is imperative to employ an adjunct dietary approach to enhance the tissue’s response to resistance exercise [3].

In nutrition as a profession, a profound comprehension of these physiological processes holds paramount significance as it endeavors to achieve goals centered around optimizing an individual’s physical condition. The selection of protein-based dietary supplements hinges on critical factors, including amino acid composition, supplement digestibility, and ease of absorption, which enhance the chosen supplement’s practicality [4]. Notably, supplements containing essential branched-chain amino acids (BCAAs) have been instrumental in modulating muscle protein biosynthesis. This influence stems from their ability to stimulate glutamine and alanine synthesis, thereby impacting resistance to training and the synthesis of proteins integral to myofibril formation.

Furthermore, the content of leucine within these supplements assumes a pivotal role as a signaling nutrient. Leucine is proficient in eliciting the release of growth hormone and insulin secretion, both of which orchestrate the metabolic conversion of carbohydrates into glycolysis to generate energy, thus facilitating the execution of resistance exercise. Additionally, it exhibits anti-catabolic properties during periods of muscle atrophy [5].

According to Hsu et al. [6], meat supplements have many advantages compared with other protein sources due to the high absorbability and bioavailability of nutrients, including essential amino acids, some complex B vitamins (B6 and B12), and important trace elements (Fe, Zn, and Zn). Regarding whey protein supplementation, reports indicate that consuming whey protein hydrolysate, known for its elevated leucine content compared with other supplements, before and after workouts enhances strength and hypertrophy, fostering improved muscle function [7]. On the other hand, the supplements market also offers vegan options; these supplements commonly contain soy hydrolysates or isolates. The imperative lies in meticulously examining athletes’ capacity to adhere to a vegan diet without compromising performance, adaptation, or recovery. There is a paucity of empirical evidence comparing the ramifications of vegan and omnivorous diets on athletes’ adaptive processes and performance metrics. This scarcity challenges drawing precise conclusions regarding the comprehensive outcomes of adopting a vegan or vegetarian diet [8].

Incorporating proteins derived from different sources, such as animal-based (e.g., meat) and plant-based (e.g., BCAAs and vegan alternatives), enables a specific exploration of potential disparities in nutritional benefits, bioavailability, or physiological responses. This methodological approach seeks to elucidate the multifaceted impact of diverse protein sources on outcomes such as body weight, body mass index (BMI), and other relevant anthropometric parameters. Furthermore, it helps to adapt individual preferences and dietary constraints to formulate more inclusive and personalized nutritional guidelines. Hence, this study aimed to assess the effects of four distinct protein-source-based supplements in young female students, specifically focusing on understanding how the combination of resistance exercise and supplementation impacts muscle growth and associated parameters. We employed bioelectrical impedance to ascertain anthropometric measurements, a non-invasive technique for assessing body composition [9].

## 2. Materials and Methods

### 2.1. Selection of Participants

Initially, a virtual conference was conducted with 30 prospective participants to elucidate the study’s objectives and to be randomly assigned to one of the five groups of this investigation. Regrettably, only two individuals declined to participate. Subsequently, the remaining 28 individuals were randomly assigned to three groups, with six participants in each group, except for two groups with five participants each. Throughout the study, one participant sustained a muscular injury, while another encountered a dermatological issue, rendering them ineligible for inclusion in the final evaluations. As a result, our final dataset (25 participants) comprised five willing participants who provided informed consent following NOM-012-SSA3-2012 [10], since one participant refused to sign this form. This consent included their commitment to strictly adhere to prescribed dietary and exercise regimens, facilitating the subsequent measurement of relevant parameters for comprehensive evaluation.

The cohort of participants consisted of young Mexican women who met specific criteria, including (1) an age range of 18–25 years, (2) a height range between 155 cm and 175 cm, and (3) a body mass index (BMI) within the standard range (18.5–24.9 kg/m^2^) as defined by the World Health Organization [11]. The selected participants had no history of prior pregnancies, were devoid of chronic pathologies, exhibited no allergic reactions to the various foods and supplements employed in the study, maintained a state of good health as documented under the NOM-004-SSA3-2012 guidelines [12], and were not undergoing any pharmacological treatments. Additionally, they consistently engaged in sporting activities. This study was conducted from September to November 2021. Participants conducted measurements at the Universidad de Monterrey (UDEM) facilities and strictly adhered to the COVID committee guidelines of the UDEM at the initiation and conclusion of the treatment. Exclusion criteria for participants were applied based on physical attributes that deviated from predefined limits, sedentary individuals with dietary restrictions, allergies, or food intolerances, and individuals unable to perform the exercises specified in the protocol. Furthermore, individuals encountering difficulties in accessing the measurement facilities were also excluded from the study.

The Universidad de Monterrey Bioethical Committee approved the study with the reference number: 05082021-N-CI (https://drive.google.com/drive/folders/1ZSmoZGt9n3yXOlBTEFLET38dodhF03vb (accessed on 14 November 2023). The ethical approval date was 5 August 2021.

### 2.2. Eating Meal Plan Design

To assess the participants’ initial energy intake (kcal), a 24 h dietary recall was conducted as described by Troncoso-Pantoja et al. [13]. Subsequently, participants’ self-reported dietary information was employed to estimate their energy consumption. Following this assessment, personalized dietary plans were devised for each participant and provided at the study’s inception, along with individualized training regimens.

The Harris–Benedict formula was applied to determine the nutritional requirements of the study cohort. Specifically, the Basal Energy Expenditure (BEE) calculation was determined for female participants using the formula reported by Hernández-Ortega et al. [14]. In addition, Total Dietary Intake (TDI) was calculated according to Ascencio Peralta [15] which considered the basal metabolic rate, the thermogenic effect of foods (10%), and physical activity (10%).

Participants were provided with personalized dietary plans based on their TDI, which included three main meals and two snacks. These dietary plans were characterized by a macronutrient distribution comprising 50–65% carbohydrates, 25–30% lipids, and protein, accounting for 10–15% of the total energy intake, as outlined by Ascencio Peralta [11]. The participants were furnished with these dietary guidelines and individualized physical training plans at the commencement of the study.

Participants were instructed to consume the designated supplement following their exercise sessions. The prescribed quantity of the supplement was determined to meet each participant’s requirements, aiming to achieve a daily intake of 1.4 g per kilogram of body weight, a quantity associated with stimulating muscle mass augmentation. It was individually calculated for each participant [16]. The administration of protein supplementation was executed through random pre-assignment to 25 participants, organized into groups of five individuals each. The groups were as follows: Group 1 received whey protein (whey protein (WP) hydrolysate, WP isolate, WP concentrate, egg albumin, milk protein concentrate, and 25 g of protein/portion) supplementation (*n* = 5), Group 2 received vegan protein (isolated of hydrolyzed protein soy, 25 g of protein/portion) supplementation (*n* = 5), Group 3 received meat protein (hydrolyzed beef protein isolate, 23 g of protein/portion) supplementation (*n* = 5), Group 4 was administered with Branched-Chain Amino Acids (BCAAs: Glycyl-Alanyl-Lysine-L-Leucine, Glycyl-Alanyl-Lysine-L-Isoleucine, Glycyl-Alanyl-Lysine-L-Valine, Glycyl-Alanyl-Lysine-L-Glutamine, 10 g of protein/portion) (*n* = 5), and Group 5 received cocoa (cocoa, <1 g of protein/portion) supplementation (control group) (*n* = 5). The supplements with different formulations were purchased at a local supermarket and given to the participants every two weeks until the end of the study, indicating the specific portion to be consumed daily to complete the individual protein requirements. For the control group, commercially available cocoa powder was given as a supplement.

### 2.3. Anaerobic Resistance Routine

Concurrently, participants engaged in two strength training regimens, each spanning four weeks, as prescribed for the study period. Detailed instructions, including visual aids to ensure the correct execution of exercises, were provided to participants. The exercise plans were structured to be carried out four days per week, with the remaining days designated for rest and recovery. The exercise routines were categorized to focus on specific muscle groups during each one-hour session, with two days dedicated to upper body muscle groups (biceps, triceps, shoulders, back, and abdomen) and two days dedicated to lower body muscle groups (quadriceps, calves, gluteals, and hamstrings). To tailor the exercise regimen to each participant’s needs, a certified sports trainer provided recommendations that could be implemented either at home or within a gym setting.

### 2.4. Anthropometric Evaluations

Before data collection, the personnel responsible for obtaining anthropometric and dietary measurements underwent comprehensive training to minimize potential biases. The body composition assessment involved using an InBody120^®^ impedance device (Microcaya S.L., Bilbo, Spain), which employed two distinct frequencies (20 kHz and 100 kHz) to perform ten measurements per participant. The following standardized procedure was employed to determine body weight (expressed in kilograms) according to Vázquez et al. [17]. Initially, the Inbody 120^®^ device was placed on a level, horizontal, and stable surface. Each participant was then weighed with minimal clothing, barefoot, after an empty bladder and a two-hour postprandial period. The participant was positioned in the device’s center, ensuring even weight distribution on both feet, with their feet placed on the electrodes. The participant faced the device, grasping the upper electrodes with both hands while their head alignment conformed to the Frankfort plane.

To assess height (in centimeters), subjects were measured without any headgear or accessories, following a specific technique. The participant stood erect with shoulders, hips, and heels in alignment, arms naturally hanging by their sides, and the head positioned according to the Frankfort plane. Participants were instructed to contract their gluteal muscles. After facing the subject, the evaluator repositioned their thumbs and index fingers close to the subject’s ears, with a separation sufficient to ensure upward traction on the mastoid processes. Subsequently, participants were asked to take a deep breath and hold it while moderate upward traction on the mastoid processes was applied. The Inbody 120^®^ stadiometer was placed at the vertex with the assistance of another trained evaluator. The Body Mass Index (BMI) was computed using the data generated by the Inbody 120^®^ device as the ratio of body weight (in kilograms) to the square of height (in square meters). The device’s interpretation recorded the resulting BMI values in kg/m^2^. Moreover, the skeletal muscle mass (SMM) and body fat mass (BFM) in kilograms were extracted from the data reported on the results sheet.

### 2.5. Statistical Analysis

Statistical analysis was conducted utilizing the SPSS version 23 software program. To assess the impact of supplements at both the study’s outset and conclusion, a paired samples t-test was employed (*p* < 0.05). Additionally, the variables were subjected to analysis through a one-way analysis of variance (ANOVA), followed by post-hoc Tukey tests (*p* < 0.05), following the methodology outlined by Yoshimura et al. [18].

## 3. Results

We assessed the effect of the consumption of diverse protein supplements derived from various sources on female participants, with a focus on key nutritional status indicators, including weight, skeletal muscle mass (SMM), body fat mass (BFM), and body mass index (BMI). Results were compared after the prescribed eight-week duration to elucidate differences observed before and after supplementation and the exercise practice.

Table 1 presents the baseline of anthropometric parameters by group of participants that consumed a specific supplement. It can be noted that for the initial measurement, SMM, BFM, and BMI did not show statistical differences. However, it was found that for the initial body weight, the groups recruited to consume the meat (Group 3) and BCAAs (Group 4) supplements showed significant differences in this parameter.

### 3.1. Eating Meal Plan and Supplementation

Table 2 shows the results obtained from a 24 h recall from all the participants. Their daily energy intake was observed to be low and did not accomplish their basal requirements. Nevertheless, due to the pandemic restrictions, a dysregulation in the population’s food consumption was reported, which was also referred to by the participants. Carbohydrate portions covered the higher amounts of energy in their diets, followed by proteins and lipids. There were statistical differences in the proteins and lipids intake between groups 4 and 5.

In the case of the calculation of the Total Energy Intake during the intervention, Table 3 presents the energy calculated for consumption by the participants. No statistical differences were found in the amount of energy necessary for the participants to meet their nutritional requirements.

Daily meal plans prescribed the same amount of energy for all the groups, even when the eating plans were personalized, and ranged from 1589.8 to 1663.2 kcal for the TEI. These results evidenced the homogeneity in the five groups regarding physiological and anthropometric parameters such as age, body weight, and height.

### 3.2. Body Composition Effects of Protein Supplementation on the Participants

**Figure 1:**
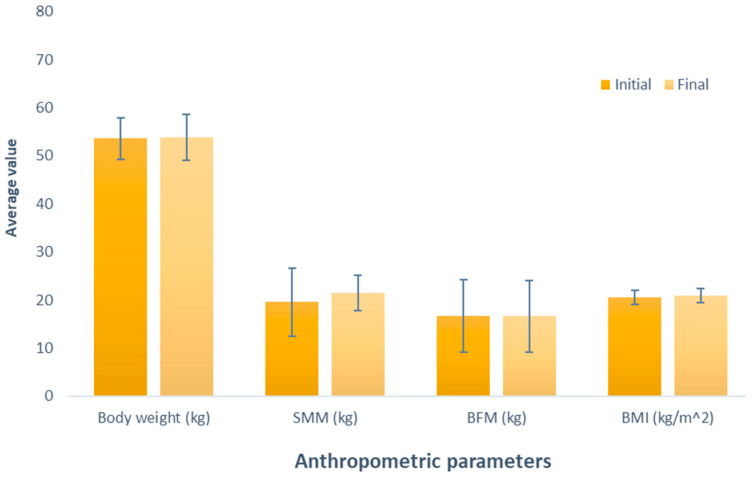
Evaluation of the effect of supplementation with whey protein. The bars represent the standard deviations. The absence of letters between bars in the same parameter denotes no statistical differences (*p* > 0.05). SMM: Skeletal muscle mass (kg); BFM: Body fat mass (kg); BMI: Body mass index (kg/m^2^).

Figure 1 depicts the effect of whey-based protein (Group 1), an increase in weight between the measurements was observed, amounting to 0.24 kg (initial weight 53.56 kg; final weight 53.8 kg); this difference was not significant (*p* = 0.534). Similarly, it was observed in the evaluation of skeletal muscle mass (SMM), where there was a non-significant (*p* = 0.337) increase of 1.84 kg (initial SMM, 19.62 kg; final SMM, 21.46 kg). Contrary to the observations in the variables, a decrease was noted when analyzing body fat mass (BFM) (initial BFM, 16.64 kg; final BFM, 16.62 kg); despite this opposite trend, the difference was also not significant (*p* = 0.980). Regarding BMI, akin to the observations in weight and SMM, there was a non-significant increase (initial BMI, 20.50 kg/m^2^; final BMI, 20.94 kg/m^2^; *p* = 0.153).

**Figure 2:**
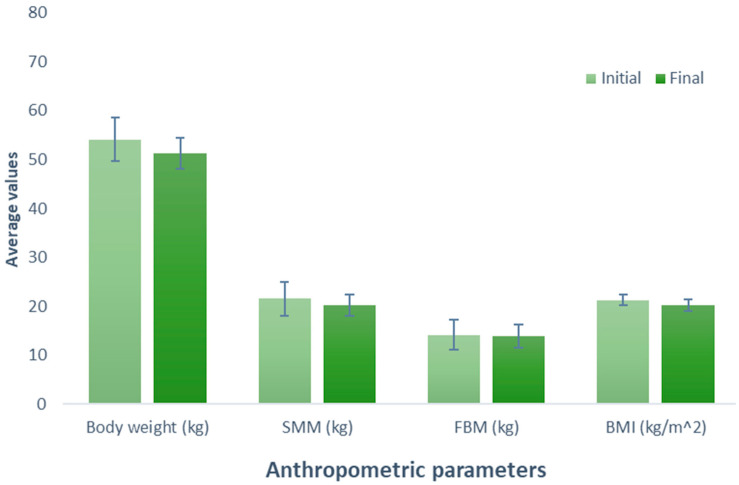
Evaluation of the effect of supplementation with vegan protein. The bars represent the standard deviations. The absence of letters between bars in the same parameter denotes no statistical differences (*p* > 0.05). SMM: Skeletal muscle mass (kg); BFM: Body fat mass (kg); BMI: Body mass index (kg/m^2^).

Examining the impact of vegan protein supplementation on the participants (Group 2), resulted in a tendency for the reduction of 2.74 kg in the body weight (initial weight, 54.08 kg; final weight, 51.34 kg) (*p* = 0.189). A parallel observation was made in the evaluation of SMM, indicating a decrease of 1.38 kg (initial SMM, 21.56 kg; final SMM, 20.18 kg) after following the exercise and supplementation treatment (*p* = 0.293). Likewise, the analysis of BFM revealed a decrease of 0.24 kg (initial BFM, 14.10 kg; final MGC, 13.86 kg; *p* = 0.785). The participants exhibited a one-unit reduction in BMI (initial BMI = 21.22 kg/m^2^ and final BMI = 20.22 kg/m^2^) (*p* = 0.196). Despite observable trends in all variables, none reached statistical significance (Figure 2).

**Figure 3:**
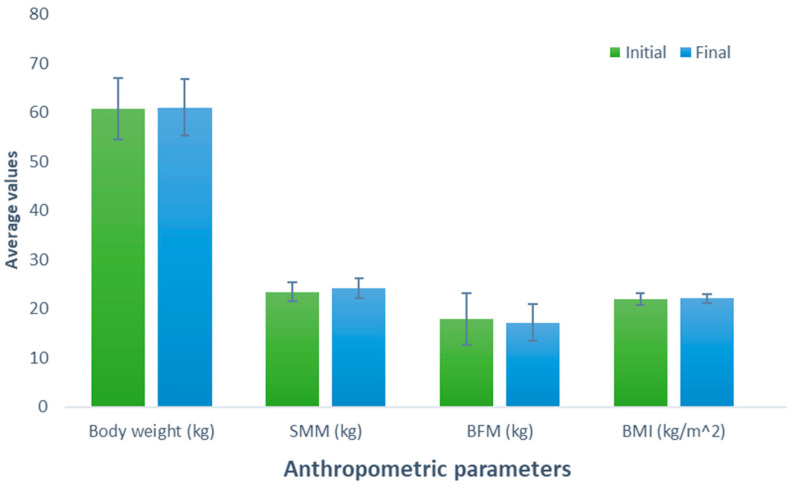
Evaluation of the effect of supplementation meat protein. The bars represent the standard deviations. The absence of letters between bars in the same parameter denotes no statistical differences (*p* > 0.05). SMM: Skeletal muscle mass (kg); BFM: Body fat mass (kg); BMI: Body mass index (kg/m^2^).

After consuming the meat protein-based supplement (Group 3), an increase of 0.26 kg was observed (initial weight, 60.78 kg; final weight, 61.04 kg) in the participants; nevertheless, this difference was not statistically significant (*p* = 0.627). Similarly, a comparable trend was observed when examining the effect of consumption of this supplement on SMM, revealing a non-significant (*p* = 0.199) increase of 0.66 kg (initial SMM, 23.44 kg; final SMM, 24.10 kg). In contrast, a non-significant decrease of 0.74 kg was noted when evaluating BFM (initial BFM, 17.86 kg; final BFM, 17.12 kg; *p* = 0.426). The BMI, body weight, and SMM parameters experienced a slight increase of 0.10 kg (initial BMI, 21.96 kg/m^2^; final BMI, 22.06 kg/m^2^; *p* = 0.600) without a significant effect (Figure 3).

**Figure 4:**
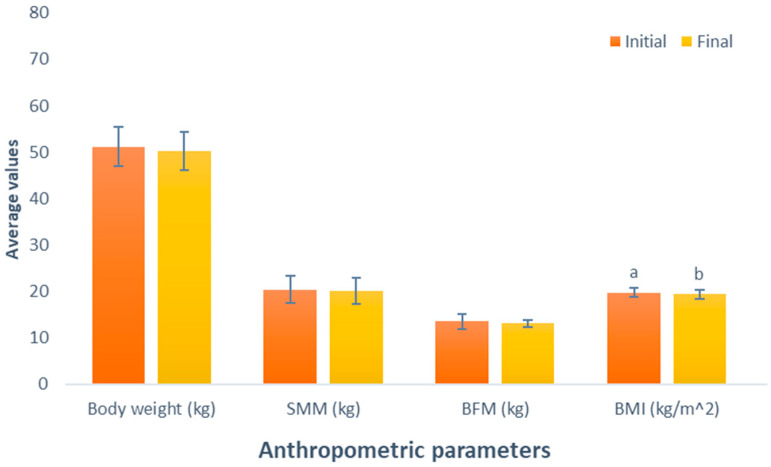
Evaluation of the effect of supplementation with branched-chain amino acids (BCAAs). The bars represent the standard deviations. Different letters between bars in the same parameter are statistically different (*p* < 0.05). SMM: Skeletal muscle mass (kg); BFM: Body fat mass (kg); BMI: Body mass index (kg/m^2^).

The results of the impact of the BCAA-based supplement consumption (Group 4) show a decline in body weight (initial weight, 51.2 kg; final weight, 50.2 kg; *p* = 0.055). This was similarly noted in SMM, and BFM displayed decreases following the administered supplementation (initial SSM, 20.4 kg; final SMM, 20 kg; initial BFM, 13.5 kg; final BFM, 13.1 kg). Unfortunately, these variables exhibited changes that were not statistically significant (*p* = 0.226 and *p* = 0.348, respectively). However, a statistically significant reduction was observed in the BMI of this group participants between the initial and final parameters (initial BMI, 19.7 kg/m^2^, final BMI, 19.4 kg/m^2^; *p* = 0.049), presented in Figure 4.

**Figure 5:**
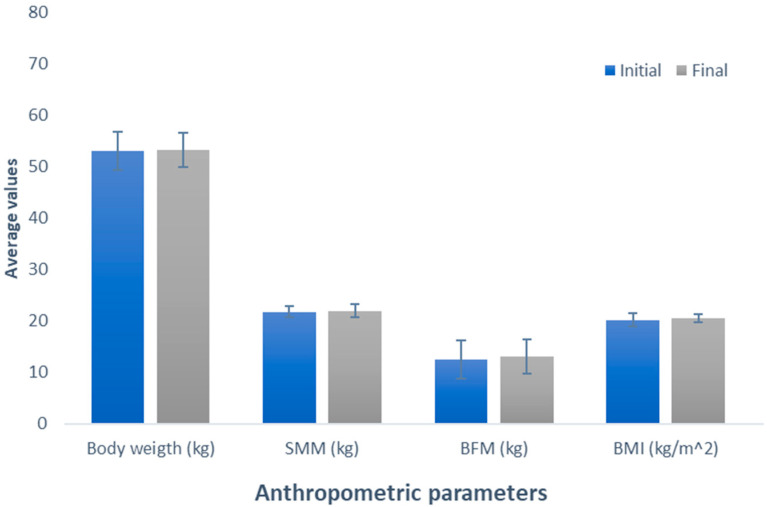
Evaluation of the effect of placebo (cocoa) supplementation. The bars represent the standard deviations. The absence of letters between bars in the same parameter denotes no statistical differences (*p* > 0.05). SMM: Skeletal muscle mass (kg); BFM: Body fat mass (kg); BMI: Body mass index (kg/m^2^).

Finally, in Figure 5, the results for the control group (Group 5), a placebo product based on cocoa, characterized nutritionally by its low carbohydrate content, was employed.

When examining the body weight variable, a non-significant increase of 0.2 kg was observed (initial weight, 53.06 kg; final weight, 53.26 kg), with a *p*-value of 0.655. Also, the SMM showed an increase of 0.16 kg (initial SMM, 21.78 kg; final SMM, 21.94 kg), a change that, despite being observed, did not reach statistical significance (*p* = 0.661). In terms of the BFM variable, like the previous variables, there was a non-significant increase of 0.56 kg (initial BFM, 12.48 kg; final BFM, 13.04 kg; *p* = 0.158). BMI exhibited a similar trend to the other variables, presenting a non-significant increase of BMI, 0.3 kg/m^2^ (initial BMI, 20.24 kg/m^2^; final BMI, 20.54 kg/m^2^; *p* = 0.281).

**Figure 6:**
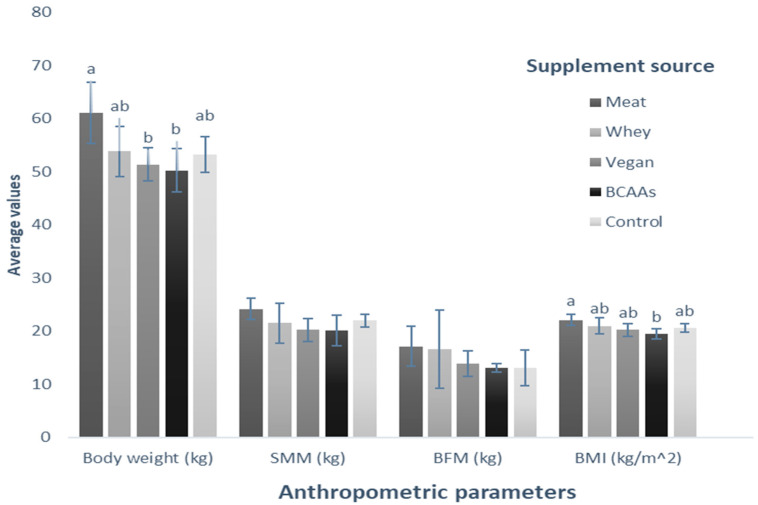
Comparison of the average value of the anthropometric parameters for each group supplemented with different protein sources. The bars represent the standard deviations of the means compared by a one-factor, post-hoc Tukey ANOVA (*p* < 0.05). Different letters between bars in the same parameter are statistically different (*p* < 0.05). SMM: Skeletal muscle mass (kg); BFM: Body fat mass (kg); BMI: Body mass index (kg/m^2^).

Additionally, we conducted a comparative analysis of the assessed variables for each treatment following an eight-week application period to discern variations in the impact of the supplements. Regarding body weight, there were no significant differences between BCAAs, vegan, control, and whey supplementation (50.24 kg, 51.34 kg, 53.26 kg, and 53.8 kg, respectively; *p* = 0.696). However, meat supplementation increased body weight compared with that recorded in subjects who consumed BCAAs and vegan supplements (*p* < 0.05). In the case of BMI, the variables behaved similarly between BCAAs (19.4 kg/m^2^), vegan (20.22 kg/m^2^), control (20.54 kg/m^2^), and whey (20.94 kg/m^2^) supplements, presenting no significant differences among them (*p* = 0.227). The same tendency was observed between the meat-based supplement (22.06 kg/m^2^) and the rest of the supplements (*p* = 0.107), except for BCAAs supplementation, where the BMI of people in this group is significantly lower than that of people supplemented with meat-based protein (*p* < 0.05). When comparing the treatments for the variables of SMM (*p* = 0.127) and BFM (*p* = 0.537), no significant differences were observed (Figure 6).

## 4. Discussion

The dietetic evaluation showed a low energy intake (kcal) by the participants before the study started, which was evidenced by the results of the 24 h recall. These results are significantly lower than those reported in a study conducted on young women aged 18–26 who reported consuming 1480 kcal in their diets, and a high percentage (85.8%) of the participants showed insufficient energy intake [19]. Considering that the participants in this study were students, another explanation for their low energy intake could be attributed to sleep efficiency. Hashimoto et al. [16] reported a deficient total energy intake in Japanese women (18–27 years), which was related to low sleep efficiency. Students frequently commented on low sleep hours due to excessive academic activities during the follow-up interviews performed during our study. No significant differences were found in lifestyle characteristics, physical activity, and dietary habits.

After the baseline analysis from the anthropometric measurements (Table 1), we observed a similar baseline across experimental groups, which is a crucial practice in experimental design to enhance internal validity and the study’s ability to make causal claims about the effects of the intervention. Moreover, the two groups presented significant differences in the initial body weight. Body weight is a comprehensive measure of mass, whereas body composition scrutinizes the body’s distinct elements, encompassing fat, muscle, bone, and water [20]. While body weight may offer a broad indication of an individual’s size, analyzing body composition provides a more intricate comprehension of the body’s constitution and is frequently regarded as a superior indicator of overall changes in our study.

Subjects who incorporated meat-based supplementation exhibited a trend to present a minor uptick in body weight and BMI, although these changes did not attain statistical significance. Notably, this observation aligns with a longitudinal cohort study conducted by Bes-Rastrollo et al. [21], which unearthed a substantial link between weight gain and red meat consumption, highlighting the contrasting findings between these studies. On the other hand, this study showed that the group with meat-based supplementation (Group 3) did not significantly increase SMM as expected, contrasting with those reported by López-Luzardo [22], where a decrease in muscle mass was demonstrated in adults when consuming hyper-protein diets. Conversely, the outcomes within the group receiving meat-based supplementation exhibited a non-significant reduction in body fat mass, mirroring findings from a study by Daly et al. [23]. Their research underscores the positive impact of a protein-enriched diet, lean red meat consumption, and the integration of progressive resistance training in enhancing lean tissue mass and muscle strength. In the case of whey-based protein (Group 1), after the eight weeks established for the study, there were increases in body weight, SMM, and BMI, along with a decrease in BFM, all of which were statistically insignificant. In this sense, Lynch et al. [24] mentioned that in a 12-week study, significant changes in body mass and muscle thickness were obtained for the initial value, which differs from the results observed in the eight weeks of this work. However, the trend in the proposed changes after eight weeks has the possibility of becoming significant, like what was reported by the authors, given that the treatment time would have been longer. Mobley et al. [25] reported no significant changes in total body mass after 12 weeks. They were significant in total body muscle mass, similar to the results of our study.

Relatedly, Nabuco et al. [26] mentioned in a 12-week study conducted on older women that changes in skeletal muscle mass were similar between groups. However, after the supplementation phase, a significant increase was found in groups that consumed whey protein before resistance exercise (RT), placebo before and after RT, and whey protein after RT compared with the control. Although comparable in the tendency to increase the evaluated parameters in the present investigation, these results were not significantly affected. On the other hand, when supplemented with vegan protein, a decrease in body weight, skeletal muscle mass, body fat mass, and body mass index was observed without statistical difference. This may be related to the differences in digestibility, bioavailability, and biological value of plant protein compared with animal origin. Bioavailability and digestibility are essential because they affect the ability of the protein source to be used in the body, which may be a factor in other supplements; there is evidence of differences in biodigestibility between proteins of plant and animal origin [27,28]. This is evidenced in a study where dairy protein digestibility is higher than soy, pea, and wheat proteins; therefore, plant-based proteins are less absorbed [29]. Furthermore, Banaszek et al. [30], in an eight-week study, observed the effects of protein supplementation of animal and plant origin, where no significant changes were found in the different groups, which coincides with what was observed in this research. Despite changes in skeletal muscle weight, body fat mass, and body mass index, these are not statistically significant.

In contrast, Hevia-Larraín et al. [31] conducted a 12-week study comparing the impact of animal and vegetable proteins in conjunction with a resistance exercise program. Both groups exhibited augmented muscle mass and strength, utilizing a protein requirement of 1.6 g/kg, with a notable increase in supplementation observed in the cohort employing vegetable protein. This differs from the outcomes of the present investigation, wherein the elevation in protein requisites and the necessity for plant-based protein were not considered. Although statistically non-significant, the alterations observed in this study may be attributed to the quality and bioavailability of vegan protein.

Specific findings stand out when contrasting and analyzing the impact of BCAAs as a supplement and their effects. For instance, Stout et al. [32] conducted a study involving ingesting a BCAA-containing supplement over three weeks of training, noting an enhancement in participants’ performance and lean body mass, including muscle mass equivalent (MME). This contrasts with the outcomes of the present evaluation, where a decrease was observed in all variables, including MME, albeit statistically non-significant. Conversely, Blomstrand and Saltin [33] undertook a study with individuals consuming a dose of a BCAA-based supplement during their training sessions, suggesting an anabolic effect of these amino acids on muscle protein metabolism during the recovery period, promoting synthesis and reducing degradation. In our study, a contrasting trend emerged, showing a tendency for a decrease in SMM and BMI, with the latter reaching statistical significance (*p* = 0.049). VanDusseldorp et al. [34], in a study involving healthy individuals engaged in resistance exercise and following a diet providing 1.2 g/kg/day of protein, supplemented this controlled protein regimen with BCAAs supplementation. The result was decreased pain perception among individuals engaged in resistance exercise. However, their findings indicated that the timing of protein consumption did not exert a discernible impact on muscle function. This emphasizes that, while adhering to a controlled protein diet with an increased amino acid intake, the influence of these amino acids on muscle recovery remains marginal. Consequently, no observable effects on muscle synthesis or strength enhancement were evident, aligning with the overarching trend observed in the study results.

It is imperative to scrutinize the impact of supplements compared with a placebo (cocoa) chosen for this purpose. In a parallel vein, a study by Mobley et al. [25] randomly allocated a sample of college-age men into groups to ingest leucine supplements, whey protein concentrate, hydrolysate, soy protein concentrate, and a placebo group utilizing maltodextrin. No differences were noted in body composition variables, such as skeletal muscle mass or strength. This lack of disparity could potentially be attributed to the relatively brief intervention duration of 12 weeks, which is four weeks longer than the timeframe of the current project. Contrary to another investigation [35], cocoa was employed to assess its effects on specific markers such as muscle damage, oxidative stress, and physical fitness in footballers. Results indicated that cocoa consumption may contribute to maintaining optimal physical condition, potentially suggesting an increase in proteins within skeletal muscle mass, akin to the growth trend observed in the present study. This observed trend could indicate a potential adaptation to resistance training, possibly facilitated by storing endogenous carbohydrates [36]. Jonvik et al. [36] conducted a study involving 60 men who ingested a casein protein supplement or an isoenergetic carbohydrate placebo over 12 weeks. The primary observation was an increase in muscle endurance, complemented by an evaluation of lean mass in the legs, revealing a significant increase with casein protein supplementation compared with the placebo group. Consequently, this study’s potential augmentation in variables can be predominantly attributed to skeletal muscle mass and proteins facilitated by protein supplementation, although statistical significance was not observed. Simultaneously, measurements conducted with an InBody 230^®^ for body composition underscore the challenge of definitively determining whether supplementation can yield a significant effect due to its short-term impact on skeletal muscle mass and other variables.

Kritikos et al. [37] evaluated a limited sample of 10 football players supplemented with whey protein, soy protein, and an isoenergetic placebo group (maltodextrin). Their conclusion suggested that a protein intake of 1.5 g/kg/day could enhance post-exercise performance in speed resistance. However, the present study did not reveal any statistical significance in the variables assessed. It is plausible that the observed trends in weight, skeletal muscle mass, and body mass index may be attributed to a protein supplementation of 1.4 g/kg/day, which is lower than the quantity proposed by those authors. The short time-lapse of the study did not allow us to observe significant changes. Similarly, Tapia López [38] explored the impact of milk with cocoa as a beverage in endurance sports, noting that this combination serves as a rich source of protein, lipids, amino acids, vitamins, and minerals. Moreover, when combined with protein, cocoa, as a carbohydrate source, may contribute to muscle recovery, enhancing participant performance. Noteworthy is the scarcity of evidence from studies conducted on women, as most research focuses on male athletes. Hence, the present study offers a potential avenue for novel findings in future research, particularly in nutritional consultation and sports nutrition.

The impact of supplements on the muscle mass of women engaged in an eight-week exercise regimen exhibited variations in parameters dependent on the type of supplement administered. Notably, the increase in weight was more pronounced in women supplemented with BCAAs and the vegan supplement, in contrast to those supplemented with whey protein. This finding aligns with the observations made by Valenzuela et al. [39], suggesting that whey and meat-based protein supplementation yield similar outcomes in terms of required protein intake and body composition, distinguishing them from a non-supplemented group. The meat-based supplement demonstrated no significant divergence in the current investigation compared with the control group receiving isocaloric carbohydrate supplementation. This observation resonates with a study by Valenzuela et al. [40], wherein a similar comparison revealed no statistically distinguishable outcomes between the two treatments. Notably, those authors reported enhancements in parameters such as *vastus lateralis* muscle thickness, thigh cross-sectional area, and the testosterone-cortisol ratio. No disparities in hematological parameters, body mass, or skinfold thickness were detected. Naclerio et al. [41] proposed that protein from meat, as consumed by male triathletes, promotes superior thigh muscle mass and iron metabolism compared to whey protein and carbohydrate-based control. This inclination was mirrored in the present study, where the meat-based supplement showed greater weight and potential mass significance, contrasting with whey protein.

Moreover, they suggest a tendency toward increased muscle mass, a trend observed across various treatments in this study. However, the duration of the application may have influenced the absence of further significant changes. Contrary to evidence suggesting hydrolyzed whey protein and casein induce protein synthesis, the present study reveals no significant difference in body weight compared with meat protein [42]. Biodigestibility is proposed as a contributing factor, where better-digested proteins supply the necessary amino acids for metabolic activities focused on synthesizing and degrading skeletal muscle [43]. Reports indicate that proteins of vegetable origin have lower digestibility (56–67 on a scale of 0–100, where 100 corresponds to full protein utilization) compared with those of animal origin (73–94), potentially explaining the observed weight difference between individuals supplemented with meat and those with vegan supplements in the present study [44].

Regarding BCAAs, it is noteworthy that specific amino acids, such as leucine, play a pivotal role in protein synthesis by acting as signaling molecules actively involved in metabolic processes. Supplementation with these amino acids contributes to a protein balance, either inducing synthesis or degradation. However, this finding appears to contradict the outcomes of the current study, where individuals supplemented with BCAAs experienced a decrease in BMI compared with those receiving meat-based supplementation [34]. On the contrary, Roelof and Smith-Ryan [45] assert that BCAA supplementation between meals over 21 days increases resting metabolic rate, enhances feelings of satiety, and decreases carbohydrate intake among women. This aligns with the findings of the present study, where the BMI reduction among women supplemented with BCAAs was statistically significant compared with those receiving meat-based supplementation. This observation supports and lends credence to the obtained result.

## 5. Study Limitations

Due to the pandemic, the authors found establishing a more controlled environment to follow up on activities challenging. This may result in an inability to capture the long-term effects of a particular intervention, limiting our understanding of sustained impacts in a small sample of participants. Dietetic parameters and physical activity rely on self-reported dietary intake, which could be subject to errors due to inaccuracies in memory or the tendency to provide socially desirable responses.

## 6. Conclusions

The dietary patterns of the participants showed deficiencies in total energy consumption. Nevertheless, the distribution of macronutrients could be considered normal. Following an eight-week exercise and nutritional intervention regimen, notable distinctions emerge between individuals who received meat supplementation and those who were supplemented with BCAAs and vegan protein alternatives. These participants exhibited reduced body weight and body mass index. Nevertheless, the meat-based supplement and the other mentioned supplements did not display statistically significant variances compared with the control group. Considering this observed pattern, extending the treatment duration is recommended to ascertain potential alterations in the other treatment modalities. The study’s practical relevance is rooted in its capacity to elucidate evidence-based dietary guidelines customized for women actively participating in anaerobic resistance exercise. This contribution can advance the refinement of targeted and personalized nutritional methodologies within the fitness and exercise science domain.

## Figures and Tables

**Table 1:** Baseline comparative anthropometric analysis by group consuming different protein supplementation.

Group/Protein Source	Age (Years)	Height (cm)	Body Weight (kg)	SMM (kg)	BFM (kg)	BMI (kg/m^2^)
1. Whey protein	19.5 (1.29) a	160.25 (2.22) a	53.56 (4.30) ab	19.44 (7.0) a	16.64 (7.55) a	20.50 (1.49) a
2. Vegan	21.6 (2.30) a	158.40 (3.05) a	54.08 (4.48) ab	21.56 (3.49) a	14.10 (3.04) a	21.22 (1.11) a
3. Meat	22.25 (1.50) a	161.0 (2.94) a	60.78 (6.34) a	23.44 (1.92) a	17.86 (5.29) a	21.96 (1.21) a
4. BCAAs	22.0 (2.45) a	159.75 (5.85) a	51.20 (4.22) b	20.40 (2.99) a	13.52 (1.58) a	19.76 (0.98) a
5. Control	20.80 (2.68) a	163.60 (2.22) a	53.06 (3.67) ab	21.78 (1.08) a	12.48 (3.68) a	20.24 (1.23) a

Mean values and the standard deviations among parenthesis compared by a one-factor, post-hoc Tukey ANOVA (*p* < 0.05). Different letters in the same column are statistically different. SMM: Skeletal muscle mass (kg); BFM: Body fat mass (kg); BMI: Body mass index (kg/m^2^).

**Table 2:** Consumption of macronutrients of the participants before the supplementation intake and anaerobic resistance exercise obtained from a 24 h recall.

Group	Carbohydrates (g)	Proteins (g)	Lipids (g)	Total Energy (kcal)
1	112.5 (47.4) a	53.50 (20.43) ab	37.50 (15.29) ab	879.2 (205.9) ab
2	97.7 (29.2) a	36.03 (7.77) ab	36.92 (23.77) ab	756.7 (179.7) ab
3	128.1 (25.2) a	64.27 (22.66) ab	29.78 (5.57) ab	988.2 (110.1) a
4	113.50 (16.90) a	71.3 (24) a	39.68 (15.17) a	1081.2 (152.2) a
5	86.4 (65.6) a	31.6 (24.4) b	10.32 (10.57) b	510 (353) b

Mean values and the standard deviations among parenthesis compared by a one-factor, post-hoc Tukey ANOVA (*p* < 0.05). Different letters in the same columns are statistically different.

**Table 3:** Energy distribution of the parameters included for calculating the total energy intake by group, including protein supplementation.

Group	BEE (kcal)	TEF (kcal)	PA (kcal)	TEI (kcal)
1	1386 (58.8) a	138.6 (5.88) a	138.6 (5.88) a	1663.2 (70.5) a
2	1337.6 (44.1) a	133.76 (4.41) a	133.76 (4.41) a	1605.1 (53) a
3	1370.7 (51.1) a	137.07 (5.11) a	137.07 (5.11) a	1644.8 (61.3) a
4	1324.7 (43.2) a	132.55 (4.47) a	132.55 (4.47) a	1589.8 (52.2) a
5	1382.7 (48) a	138.27 (4.80) a	138.27 (4.80) a	1659.2 (57.6) a

Mean values and the standard deviations among parenthesis compared by a one-factor, post-hoc Tukey ANOVA (*p* < 0.05). Different letters in the same columns are statistically different. BEE: Basal Energy Expenditure; TEF: thermogenic effect of foods; PA: physical activity; TEI: Total energy intake.

## Data Availability

The datasets generated during and/or analyzed during the current study are available from the corresponding author upon reasonable request.
